# Predatory publishing continues

**DOI:** 10.1002/nop2.226

**Published:** 2018-11-22

**Authors:** Roger Watson

I keep hoping that there will be no more need to address predatory publishing and I can cover more substantive nursing and clinical issues in these editorials. However, between each editorial—and many have been on predatory publishing (Watson, 2015a,b, 2017a,b, 2018)—something new arises. A recent email from the Cabell’s team indicated that there are now over 10,000 verified predatory journals and some suspicion that this could be the “tip of the iceberg.” It is hardly surprising, therefore, that people continue to be defrauded by these criminals who have polluted academic publishing. I have recently returned from the Middle East where I met someone who thought they were publishing in a nursing journal with an impact factor of 14. He was crestfallen when I told him that no nursing journal on the Clarivate list had an impact factor exceeding 4. He had parted with several hundred US dollars for the privilege of being published in a predatory journal. But his problems did not stop there. He asked the journal not to publish his article and, assuming they had agreed, he proceeded to publish the article in a *bona fide* journal only to be accused of duplication when he came up for annual review. The article had, unknown to him, been published by the predatory journal. I do not know where all this will end for him but predatory journals are notoriously unresponsive to emails unless you are offering them a manuscript and, of course, money.

Since my last editorial, I was very disappointed to learn that, in the UK, the government funded Research Excellence Framework (REF) will not discriminate against predatory journals. REF is a regular mechanism whereby research outputs, mainly in the form of published articles, are assessed for quality. Funding is then distributed to UK universities on that basis. The REF process is done by peer review and is not done, as with the Excellence in Research Australia (ERA), on the basis of journal quality or any other metrics (although citation information is made available). ERA uses a pre‐determined list of journals ranked according to their quality, but REF considers the contents of the articles, and this is the excuse being given for being unable to discriminate against predatory journals. I have to say that this information regarding REF is not published but comes to me from a very reliable source. I am disappointed because it seems, in the UK, we have simply given up against the onslaught of the predatory publishers and given individual academics, who may wish to take this route with little regard for their reputation, and excuse to publish in predatory journals. This may become more common as we reach the final stages of the REF assessment period in 2020, then the lure of rapid publication without the barriers of peer review seem too tempting to resists for academics without the required number of articles. I hope this is not going to be a permanent decision by those responsible for REF in the UK and that we can look forward to a day when journal quality does count for something in this important exercise, one which is used internationally to rank universities.
